# Optimized Application of ^68^Ga-Prostate-Specific Membrane Antigen-617 Whole-Body PET/CT and Pelvic PET/MR in Prostate Cancer Initial Diagnosis and Staging

**DOI:** 10.3389/fmed.2021.657619

**Published:** 2021-05-13

**Authors:** Chunxia Qin, Yongkang Gai, Qingyao Liu, Weiwei Ruan, Fang Liu, Fan Hu, Xiaoping Zhang, Xiaoli Lan

**Affiliations:** ^1^Department of Nuclear Medicine, Union Hospital, Tongji Medical College, Huazhong University of Science and Technology, Wuhan, China; ^2^Hubei Key Laboratory of Molecular Imaging, Wuhan, China; ^3^Department of Urology, Union Hospital, Tongji Medical College, Huazhong University of Science and Technology, Wuhan, China

**Keywords:** ^68^Ga-PSMA, PET/CT, PET/MR, benign prostatic hyperplasia, prostatitis, prostate cancer

## Abstract

**Purpose:** To analyze ^68^Ga-PSMA-617 PET/CT or PET/MR and delayed PET/MR images in patients diagnosed with or suspicion of prostate cancer, and to explore the optimal use of PET/CT and PET/MR for initial diagnosis and staging in prostate cancer.

**Methods:** Images from conventional scan by ^68^Ga-PSMA whole-body PET/CT or PET/MR followed by delayed pelvic PET/MR were retrospectively analyzed. Prostatic ^68^Ga-PSMA uptake was measured as SUVmax1 (conventional scan 1 h post injection) and SUVmax2 (delayed scan 3 h post injection). Age, PSA levels, and SUVmax were compared between benign and malignant cases. The correlation of SUVmax1 and SUVmax2 was analyzed. Diagnostic performance was evaluated by ROC analysis.

**Results:** Fifty-six patients with 41 prostate cancers and 15 benign prostate lesions were enrolled. Fifty-three patients had paired conventional and delayed scans. Age, tPSA, fPSA levels, and SUVmax were significantly different between benign and malignant cases. A good correlation was found between SUVmax1 and SUVmax2. There was significant difference between SUVmax1 and SUVmax2 in the malignant group (*p* = 0.001). SUVmax1 had superior diagnostic performance than SUVmax2, SUVmax difference and PSA levels, with a sensitivity of 85.4%, a specificity of 100% and an AUC of 0.956. A combination of SUVmax1 with nodal and/or distant metastases and MR PI-RADS V2 score had a sensitivity and specificity of 100%. Delayed pelvic PET/MR imaging in 33 patients were found to be redundant because these patients had nodal and/or distant metastases which can be easily detected by PET/CT. PET/MR provided incremental value in 8 patients at early-stage prostate cancer based on precise anatomical localization and changes in lesion signal provided by MR.

**Conclusion:** Combined ^68^Ga-PSMA whole-body PET/CT and pelvic PET/MR can accurately differentiate benign prostate diseases from prostate cancer and accurately stage prostate cancer. Whole-body PET/CT is sufficient for advanced prostate cancer. Pelvic PET/MR contributes to diagnosis and accurate staging in early prostate cancer. Imaging at about 1 h after injection is sufficient in most patients.

**ClinicalTrials.gov****:** NCT03756077. Registered 27 November 2018—Retrospectively registered, https://clinicaltrials.gov/show/NCT03756077.

## Introduction

Prostate cancer is a common malignancy harmful to health of old males. The incidence ranks second (13.5%) and it is the fifth leading cause of cancer death (6.7%) among males worldwide (1). Accurate diagnosis and staging are very important to choose the most suitable treatment.

Serum prostate-specific antigen (PSA) testing and digital rectal examination (DRE) are the most commonly used initial screening methods for prostate disease (2). The limitations of PSA level as a prostate cancer biomarker are well-known because false positive and false negative results are common, and screening for prostate cancer with PSA is generally no longer recommended (3). The value of DRE is limited in the early stages of the disease. Systematic transrectal ultrasound (TRUS)–guided biopsy is regarded as a standard, but it has frequent false-negative results and underestimates the final Gleason score of the tumor compared with histologic examination after radical prostatectomy (4).

Imaging technologies play an important role in the management of prostate cancer. Conventional imaging modalities, including ultrasound, computed tomography (CT), magnetic resonance imaging (MRI), and bone scan are commonly used in the diagnosis, staging, and restaging of prostate cancer (5). However, these conventional imaging modalities are usually regional imaging, or have limited accuracy in small lymph node metastases and small-volume bone metastases. Molecular imaging is regarded as a promising approach to improve prostate cancer diagnosis and staging (6). Several positron emission tomographic (PET) tracers, like ^18^F-FDG, ^18^F-fluorocholine, ^11^C-choline, and ^11^C-acetate, have been studied in patients with prostate cancer, but the diagnostic performance of most PET radiotracers has so far remained limited (7, 8).

Prostate-specific membrane antigen (PSMA) is a transmembrane protein which is overexpressed in prostate cancer (9, 10). It becomes a promising target for specific imaging of prostate cancer (11). In recent years, gallium 68 (^68^Ga) has been used to label PSMA ligands for PET imaging (12). Initial experience using ^68^Ga-PSMA PET/CT indicates that ^68^Ga-PSMA PET can visualize relapses and metastases of prostate cancer with high contrast through binding to the extracellular domain of PSMA and internalization (13). Studies have described the superior value of ^68^Ga-PSMA ligand PET imaging to conventional imaging in different clinical scenarios, including differential diagnosis; guiding biopsy, surgery and radiotherapy; initial staging and restaging; recurrence detection and selecting patients who may benefit from systemic targeted radionuclide therapy (14–16).

PET/MR is a hybrid technology that can provide both biologic and morphologic information. Recently introduced ^68^Ga-PSMA PET/MR combines multi-parametric MRI (mpMRI) along with molecular information of PSMA expression into a “one-stop shopping” procedure for better anatomic localization and characterization of prostate lesions. Compared with PET/CT, simultaneous PET/MR has the advantages of reduced radiation exposure and inherent higher soft-tissue contrast resolution (17, 18). PSMA PET/MR is particularly important for accurate localization and assessment of the extent of pelvic disease in the initial staging of prostate cancer. Despite the advantages of PSMA PET/MR over PSMA PET/CT, cost, scanning time, and patient comfort should also be considered. Domachevsky et al. (19) demonstrated that although early PET/MR has very good agreement compared to same-day PET/CT, PET/CT, and early PET/MR cannot be used interchangeably. They further demonstrated that pelvic PSMA PET/MR is better than whole-body PSMA PET/CT for detecting extensions of localized disease, and may be useful for initial evaluation of prostate cancer (20).

Some PET centers have both PET/CT and PET/MR. Does every patient need to undergo both PET/CT and PET/MR? How to choose the right scan is a matter of concern. The purpose of this study was to retrospectively analyze the images of whole-body PSMA PET/CT or PET/MR followed by delayed limited pelvic PSMA PET/MR in patients diagnosed with or suspicion of prostate cancer, and to explore how to rationally use PET/CT and PET/MR in prostate diseases.

## Materials and Methods

### Patients

This study was approved by Institutional Review Board. Patient data involved in a study that was registered on ClinicalTrials.gov (NCT03756077) were retrospectively analyzed. Inclusion criteria include all of the following: (a) patients diagnosed with or suspicion of prostate cancer; (b) patients underwent whole-body ^68^Ga-PSMA PET/CT or PET/MR followed by delayed pelvic PET/MR; (c) patients have pathological results or follow-up results, systemic multi-point biopsy of prostate was performed 1 month prior PET imaging or after PET imaging. Patients with any other malignancies and if they had been pretreated for prostate cancer were excluded. General information and biochemical test results were collected. All the patients were divided into a “malignant group” or a “benign group” according to the histological or follow-up results. Patients with prostate cancer were staged according to AJCC Prognostic Groups by combination of TNM, PSA level and Gleason score.

### Imaging Protocol

^68^Ga was produced from a ^68^Ge/^68^Ga generator (ITG GmBH, Munich, Germany) by eluting with 0.05N hydrochloric acid. ^68^Ga-PSMA-617 was synthesized using an ITG manual synthesis module as described previously (21). Briefly, 4 mL of ^68^GaCl3 was reacted with 20 μg (20 nmol) PSMA-617 ligand (Jiangsu Huayi Technology Company, Changshu, China) in 1 ml of 0.25 M sodium acetate buffer for 5 min at 105°C. The production was purified in a C18 cartridge and collected through a 0.22-μm-pore filter.

All patients were intravenously injected with a dose of 173.53 ± 50.69 MBq (4.69 ± 1.37 mCi) of ^68^Ga-PSMA-617. Patients were encouraged to drink water after injection and asked to empty their bladder before PET scan. Conventional imaging from vertex to proximal legs were performed using a hybrid PET/CT scanner (Discovery VCT; GE Healthcare, Waukesha WI, USA) or a time-of-flight (TOF) hybrid PET/MR scanner (SIGNA PET/MR; GE Healthcare). Delayed pelvic PET/MR images were acquired using hybrid PET/MR.

PET/CT acquisition followed our standard protocol. A CT scan (120 kV, 110 mAs) was acquired after a scout image with a scanning thickness of 3.75 mm, followed by whole-body emission static PET imaging in a three-dimensional (3D) mode at 3 min per bed position. PET images were attenuation-corrected using CT images, and reconstructed using an ordered-subset expectation maximization (OSEM) iterative reconstruction algorithm (28 subsets and 2 iterations) and co-registered with CT images (Xeleris; GE Healthcare).

For PET/MR, MR attenuation images were acquired using ZTE technology after acquisition of localization images. The PET acquisition of PET/MR was performed in 3D mode for 6 min per bed position (89 sections per bed) in five bed positions. MR imaging of the brain [axial T2-weighted, T1-weighted, and fluid-attenuated inversion recovery (FLAIR)] was performed along with the PET scan, then whole-body imaging (from skull base to midthigh in four bed positions, a high-resolution axial T1-weighted LAVA-Flex sequence and a coronal T2-weighted fast recovery fast spin echo [FRFSE] sequence in two planes were included) were acquired during the PET scan. Next, pelvic dedicated mpMRI images of the prostate [transverse, coronal, and sagittal T2-weighted images and diffusion-weighted spin-echo echo-planar images (b-factor, 0/1,000/1,400 s/mm^2^)] were acquired along with a 10 min per bed position PET imaging. Other MR protocols were included when clinically required. The delayed pelvic PET/MR imaging was the same to the previous pelvic PET/MR. All PET data were reconstructed with TOF information, using the system's default 3D OSEM protocol iterative reconstruction algorithms with 2 iterations and 28 subsets and co-registered to MR images on a workstation (AW, GE Healthcare).

### Image Analysis

PET/CT and PET/MR images were interpreted by two experienced nuclear medicine physicians using dedicated software on the AW workstation. Visual assessment was used for characterizing PSMA-avid lesions in axial, coronal and sagittal reconstructions. Lymph nodes, bone lesions and other foci suspected of being distant metastases were evaluated first. Intra-prostatic PSMA-avid foci were defined as PSMA uptake greater than the adjacent prostate gland or background on PET/CT or PET/MR, and regions of interest (ROIs) were manually drawn on PSMA-avid area or prostate bed if presented with a diffuse pattern of uptake, and maximum standard uptake value (SUVmax) were measured, the SUVmax in the conventional scan was defined as “SUVmax1,” and the SUVmax in the delayed scan was defined as “SUVmax2.” Capsular invasion; seminal vesicle, bladder or other adjacent organ involvement; and involvement of small pelvic lymph nodes were identified if PSMA uptake and abnormal MR signal were seen outside the boundaries of the prostate gland or corresponded to sites on PET/MR images. Version 2 of the Prostate Imaging Reporting and Data System (PI-RADS V2) was used to score prostatic regions with abnormal signal on MR images (22).

### Statistical Analysis

Independent samples *t*-tests were performed to compare the mean values between the malignant group and the benign group. Paired samples *t*-tests were performed to compare SUVmax1 and SUVmax2. All the correlations were analyzed using Spearman's rank correlation test, and a scatter diagram was drawn with the regression line. Bland–Altman plots were used to assess the agreement between SUVmax1 and SUVmax2. ROC curves were generated to assess the diagnostic performance of each parameter, and to calculate a cutoff value. The sensitivity and specificity were calculated on a per-patient basis imaging diagnosis against the final clinical diagnosis. All statistical analyses were performed using SPSS Statistics, version 22 (IBM, Armonk, NY, USA). A *p* < 0.05 was considered to indicate significant difference.

## Results

### Scanner Usage, Characteristics of Patients, and Prostatic Features in Benign and Malignant Groups

Of the 56 patients enrolled between May 2018 and January 2020. Conventional imaging began at 70.0 ± 16.9 min (PET/CT) or 74.3 ± 12.5 min (PET/MR) [*p* = 0.357] after injection. Delayed pelvic PET/MR images were acquired at 171.2 ± 37.9 min after injection. Forty patients underwent whole-body PET/CT and delayed pelvic PET/MR, 13 patients underwent whole-body PET/MR and delayed pelvic PET/MR, 3 patients underwent only whole-body PET/MR. Finally, forty-one patients were diagnosed as malignant and 15 were benign. In the malignant group, 32 patients were confirmed by histopathology, including 19 acinar adenocarcinoma, 1 ductal adenocarcinoma, 5 adenocarcinoma, and 7 prostate cancers with undefined pathological type. The other 9 patients were diagnosed by imaging and response of endocrine therapy. The benign group included benign prostatic hyperplasia (BPH) and/or prostatitis, 6 patients had biopsy results, symptoms of all patients eased and PSA level decreased after anti-inflammatory or anti-prostatic hyperplasia treatment. The follow-up time was at least 1 year. The patient characteristics are listed in Table 1.

**Table 1:** Patient characteristics and prostatic features in benign group and malignant group.

	**Benign group**	**Malignant group**	***p*-value**
Age (y)	61.93 ± 7.41 (47–75)	71.02 ± 8.58 (52–91)	*p* = 0.001
tPSA (ng/ml)	15.67 ± 18.43 (*n* = 15)	135.98 ± 232.92 (*n* = 37)^*^	*p* = 0.004
fPSA (ng/ml)	2.82 ± 4.45 (*n* = 14)	11.18 ± 11.49 (*n* = 35)^#^	*p =* 0.001
fPSA/tPSA	0.164 ± 0.086 (*n* = 14)	0.132 ± 0.840 (*n* = 27)	*p =* 0.247
Prostatic SUVmax1	4.09 ± 0.96	20.31 ± 15.74	*p* < 0.001
Prostatic SUVmax2	4.63 ± 1.34	24.53 ± 16.38	*p* < 0.001
**Location of lesion in prostate**			
Peripheral zone	7 (46.7%)	12 (29.3%)	
Central zone	5 (33.3%)	3 (7.3%)	*p =* 0.006
Peripheral zone+ central zone	3 (20.0%)	26 (63.4%)	

* *6 patients have tPSA >100 ng/ml, 2 >1,000 ng/ml*;

# *5 patients have fPSA >30 ng/ml. The limit values were used in statistics*.

**Figure 1:**
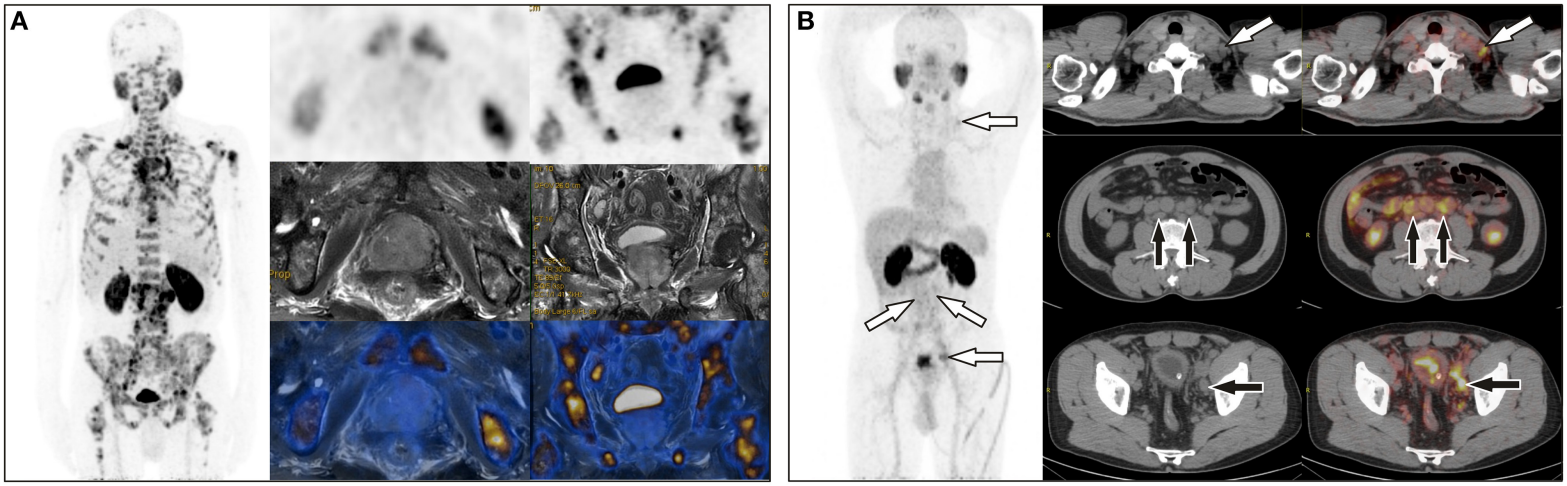
**(A)** A 65-year-old man with a total PSA level of > 1,000 ng/L and free PSA > 30 ng/L underwent ^68^Ga-PSMA PET/MR, which revealed extensive bone metastases but negative uptake of PSMA in the prostate. **(B)** A 64-year-old man with a total PSA level of 13.9 ng/L and free PSA of 2.15 ng/L underwent ^68^Ga-PSMA PET/CT, which showed uptake in multiple lymph nodes in the left supraclavicular area, retroperitoneum, and the left iliac chain, but no focal uptake in prostate. Prostate acinar adenocarcinoma with left iliac lymph node metastases (6/6) were histopathologically proved.

**Figure 2:**
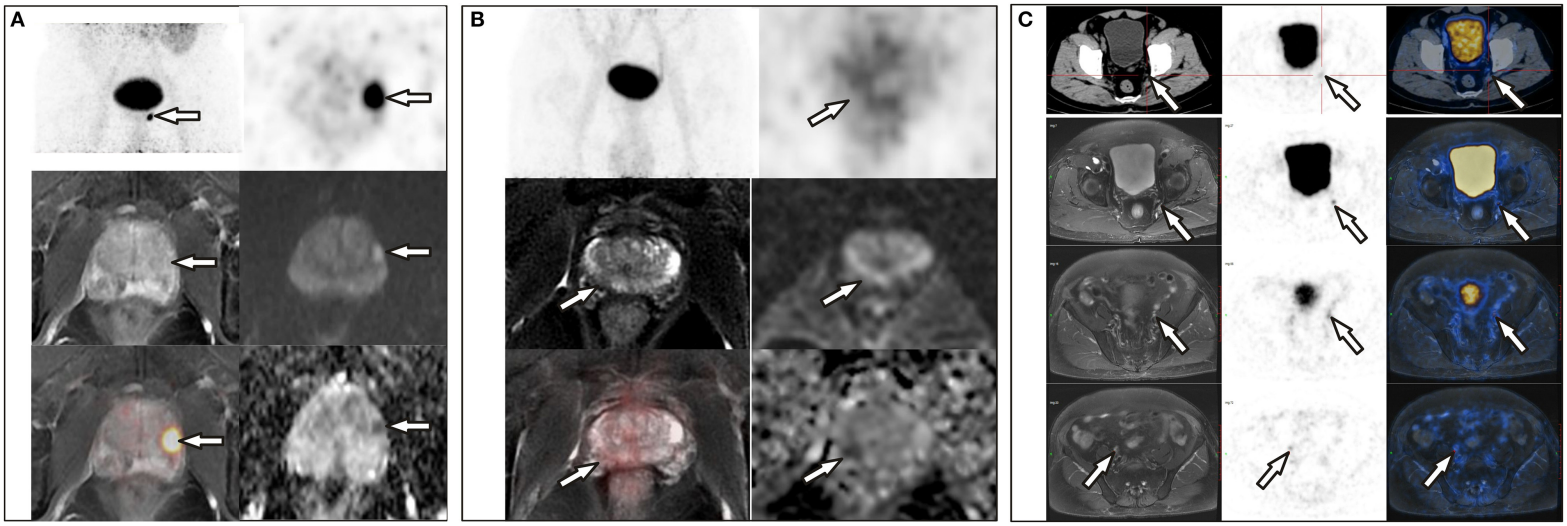
**(A)** A 72-year-old man with a PSA level of 21.26 ng/L underwent ^68^Ga-PSMA PET/CT and delayed pelvic PET/MR. Positive PSMA uptake was revealed in the left peripheral zone, consistent with the signal change in mpMR. **(B)** A 68-year-old man with proven prostate cancer in the right lobe underwent PET/MR for staging. Axial T2-weighted image shows an ill-defined hypointense lesion in the right peripheral zone with corresponding hypo-intensity on the apparent diffusion coefficient map. No significant hyperintense signal was observed on DW images (*b* = 1,000 s/mm^2^). This was assigned a PI-RADS score of 4, but negative PSMA uptake was observed with diffuse ^68^Ga-PSMA uptake in the prostate (SUVmax, 4.10). **(C)** A 71-year-old man with proven prostate cancer after prostate transurethral resection, ^68^Ga-PSMA PET/CT and pelvic PET/MR were performed for staging. PET/CT revealed only one small lymph node in the left pelvic cavity, which was revealed more clearly on PET/MR, and more lesions were revealed on PET/MR, which were proved to be metastases after surgery.

The differences in age, total prostate-specific antigen (tPSA), free prostate-specific antigen (fPSA), and prostatic SUVmax between the benign group and the malignant group were statistically significant. Intra-prostatic PSMA-avid foci were found in 6 patients in the benign group (40.0%) and 35 in malignant group (85.4%). In the benign group, three of six presented with symmetrical accumulation in posterior peripheral bands at the base of prostate. In six prostate cancer patients without intra-prostatic PSMA-avid foci, one had bone metastases, two had lymph node metastases, two had both lymph node and bone metastases. Representative images are shown in Figures 1, 2. The locations of the lesions in prostate are shown in Table 1; most were in the peripheral or central zone in the benign group. In the malignant group, most involved both the peripheral zone and central zone (Figure 2).

### Characteristics of Prostatic ^68^Ga-PSMA-617 Uptake and Its Correlation With Gleason Score and PSA Level

**Figure 3:**
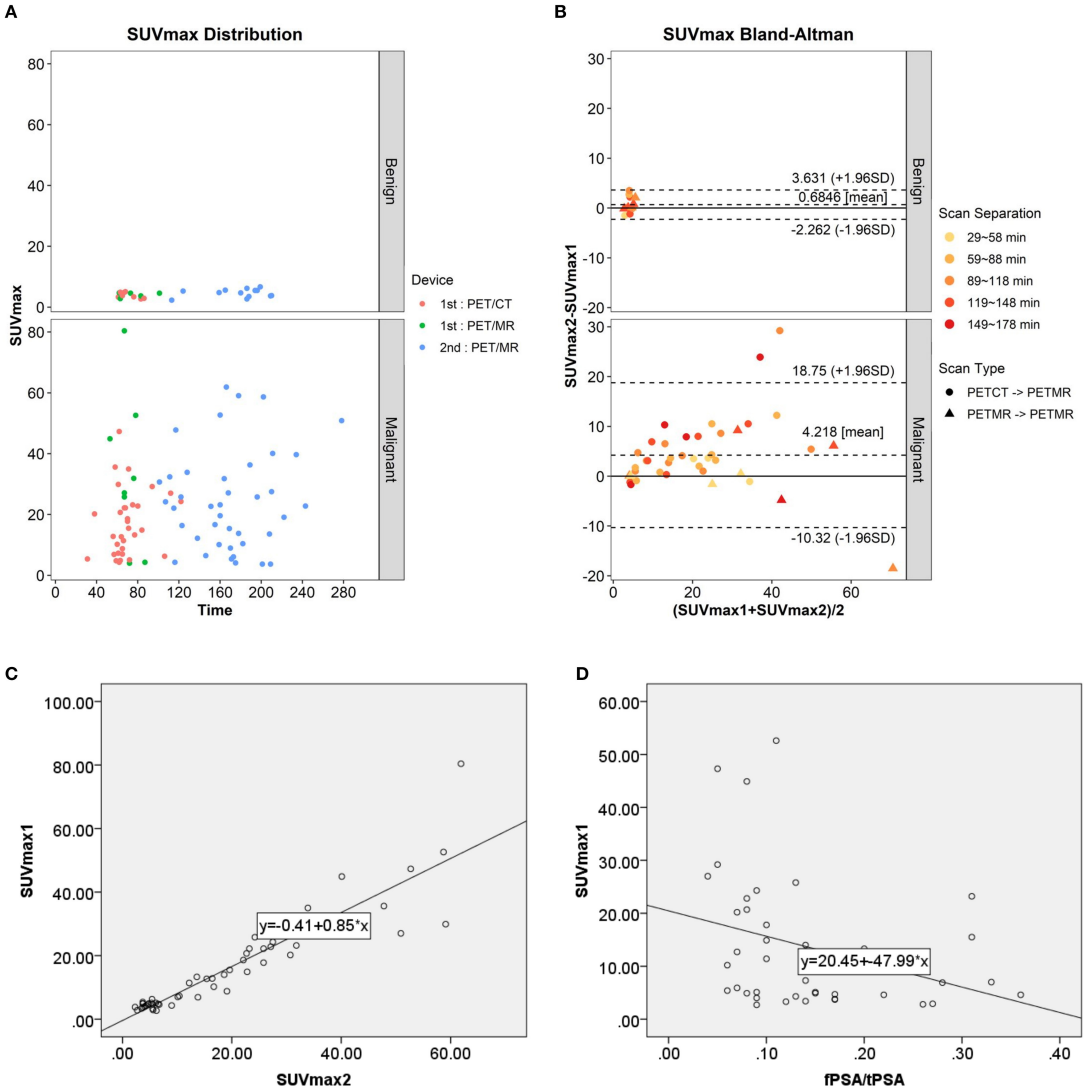
**(A)** Scatter diagram of SUVmax from different scanner at different time point. **(B)** Bland–Altman plots of the differences against the means of SUVmax1 with SUVmax2. **(C)** Scatter plot and correlation of SUVmax1 and SUVmax2. **(D)** Scatter plot and correlation of SUVmax1 and fPSA/tPSA.

In the 53 of the 56 patients with paired conventional and delayed scans, ^68^Ga-PSMA-617 uptake in the two phases was compared. In the benign group, the mean prostatic SUVmax values in the conventional and delayed scans were 3.95 ± 0.88 and 4.64 ± 1.34 (*n* = 13), respectively. No significant difference was found between the two phases (*t* = −1.642, *p* = 0.127). In the malignant group, the mean prostatic SUVmax in the conventional and delayed scans were 20.31 ± 15.74 and 24.53 ± 16.38 (*n* = 40), respectively, with a significant difference between the two phases (*t* = −3.695, *p* = 0.001). The scatter diagram of SUVmax from different scanners at different time point is shown in Figure 3A. Bland–Altman plots reveal a scatter diagram of the differences plotted against the means of SUVmax values from conventional whole-body PET/CT or PET/MR with delayed pelvic PET/MR. In the benign group, the mean SUVmax has a narrow range at low levels, and the mean difference is 0.68, with the limits of agreement (LOA) between −2.26 and 3.63. In the malignant group, mean SUVmax had a wide range, and the mean difference was 4.22, with LOA between −10.32 and 18.75 (Figure 3B). Good correlation was found between SUVmax1 and SUVmax2 [*r* = 0.932, *p* < 0.001, Figure 3C, Y(SUVmax1) = −0.41 + 0.85^*^X(SUVmax2)]. No significant correlation was found between SUVmax and Gleason score, tPSA, and fPSA. There was a significant negative correlation between SUVmax and fPSA/tPSA (*r* = 0.674, *p* = 0.039). The scatter diagram shown in Figure 3D expresses Y (SUVmax1) as the result of 20.45–47.99^*^X (SUVmax2).

### Gleason Score, AJCC Prognostic Stage and Primary Lesion, Involvement of Surrounding Tissues, and Distant Metastases in Patients With Prostate Cancer

Because this was a retrospective study, only 30 patients had available Gleason scores. The numbers of patients with different Gleason scores and AJCC prognostic stage are listed in Table 2. No significant differences were found in SUVmax among different groups according to Gleason score and AJCC prognostic stage. The higher Gleason score, the higher incidence of nodal and distant metastases. However, nodal and distant metastases also occurred even in patients with low Gleason score.

**Table 2:** Clinical and imaging characteristics in patients with prostate cancer.

**Characteristic**	**No**.	**Prostatic SUVmax**	**Seminal vesicle involvement**	Other adjacent organ involvement^*^	**Lymph node metastasis**	**Bone metastasis**	**Other metastases^#^**
Gleason score	30	*p =* 0.550	16	8	23	13	4
1 (≤6)	4		0	1 (12.5%)	2 (8.7%)	3 (23.1%)	2 (50%)
2 (3 + 4 = 7)	3	24.3 ± 22.6	2 (12.5%)	0	2 (8.7%)	2 (15.4%)	1 (25%)
3 (4 + 3 = 7)	2	(GS ≤ 7)	0	0	0	0	0
4 (4 + 4 = 8)	9	17.3 ± 12.4	6 (37.5%)	4 (50%)	8(34.8%)	2 (15.4%)	0
5 (9 or 10)	12	21.3 ± 15.9	8 (50%)	3 (37.5%)	11(47.8%)	6 (46.2%)	1 (25%)
AJCC prognostic stage	41	*p =* 0.700	23	11	29	19	7
2A	5	20.1 ± 14.8	0	0	0	0	0
2B	3		0	0	0	0	0
4A	14	19.3 ± 12.1	10 (43.5%)	3 (37.5%)	14 (48.3%)	0	0
4B	19	21.1 ± 18.9	13 (56.5%)	8 (62.5%)	15 (51.7%)	19 (100%)	7 (100%)

* *Other adjacent organ involvement include 11 bladder involvement, 1 combined with urethra and corpus spongiosum penis involvement, 1 right ureter and rectum involvement, 1 right ureter involvement, and 1 rectum involvement*.

# *Other distant metastases include 4 patients with bilateral lung metastases, 1 patients with bilateral lung metastases and liver metastases, 2 patients with muscle metastases (1 right obturator, 1 right psoas major)*.

*GS: Gleason score*.

### Diagnostic Performance

**Figure 4:**
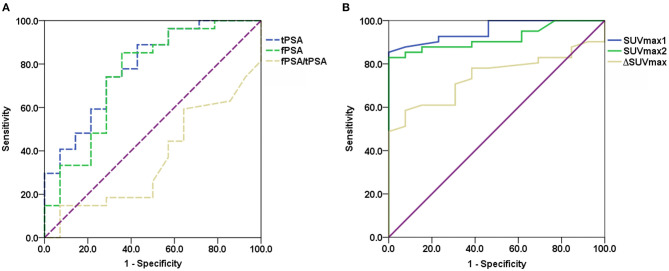
ROC curves evaluating diagnostic performance of PSA levels **(A)** and ^68^Ga-PSMA SUVmax **(B)**.

ROC analysis was performed to evaluate the diagnostic performance of tPSA, fPSA, and fPSA/tPSA (Figure 4A), SUVmax1, SUVmax2, and the difference between SUVmax1 and SUVmax2 (ΔSUVmax) (Figure 4B) for differentiating malignant from benign lesions. Cutoff values, sensitivity, specificity, AUC, 95% confidence interval (CI) and *P*-values are shown in Table 3. SUVmax1 from the conventional PET/CT or PET/MR revealed the best diagnostic performance with an AUC of 0.956, cutoff value of 5.25 (sensitivity, 85.4%; specificity, 100%; *p* < 0.001). So SUVmax1 was used for the following analysis. When combining SUVmax1 with nodal/distant metastases, the sensitivity improved to 95.1%. If we combine SUVmax1, nodal/distant metastases and MR PI-RADS V2, sensitivity and specificity both reached 100%.

**Table 3:** Diagnostic performance of several indices and its combination.

	**Cut-off**	**sensitivity**	**specificity**	**AUC**	**95% CI**	***P***
tPSA	7.73	88.9%	57.1%	0.786	0.637–0.934	0.003
fPSA	1.58	85.2%	64.3%	0.751	0.583–0.920	0.009
SUVmax1	5.25	85.4%	100%	0.956	0.907–1.000	<0.001
SUVmax2	7.85	82.9%	100%	0.919	0.848–0.991	<0.001
ΔSUVmax	0.90	70.7%	69.2%	0.742	0.614–0.870	0.009
LN/Distant metastases + SUVmax1		95.1%	100%			
LN/Distant metastases + SUVmax1+mpMR		100%	100%			

### Diagnostic Overview of All Patients in This Study

**Figure 5:**
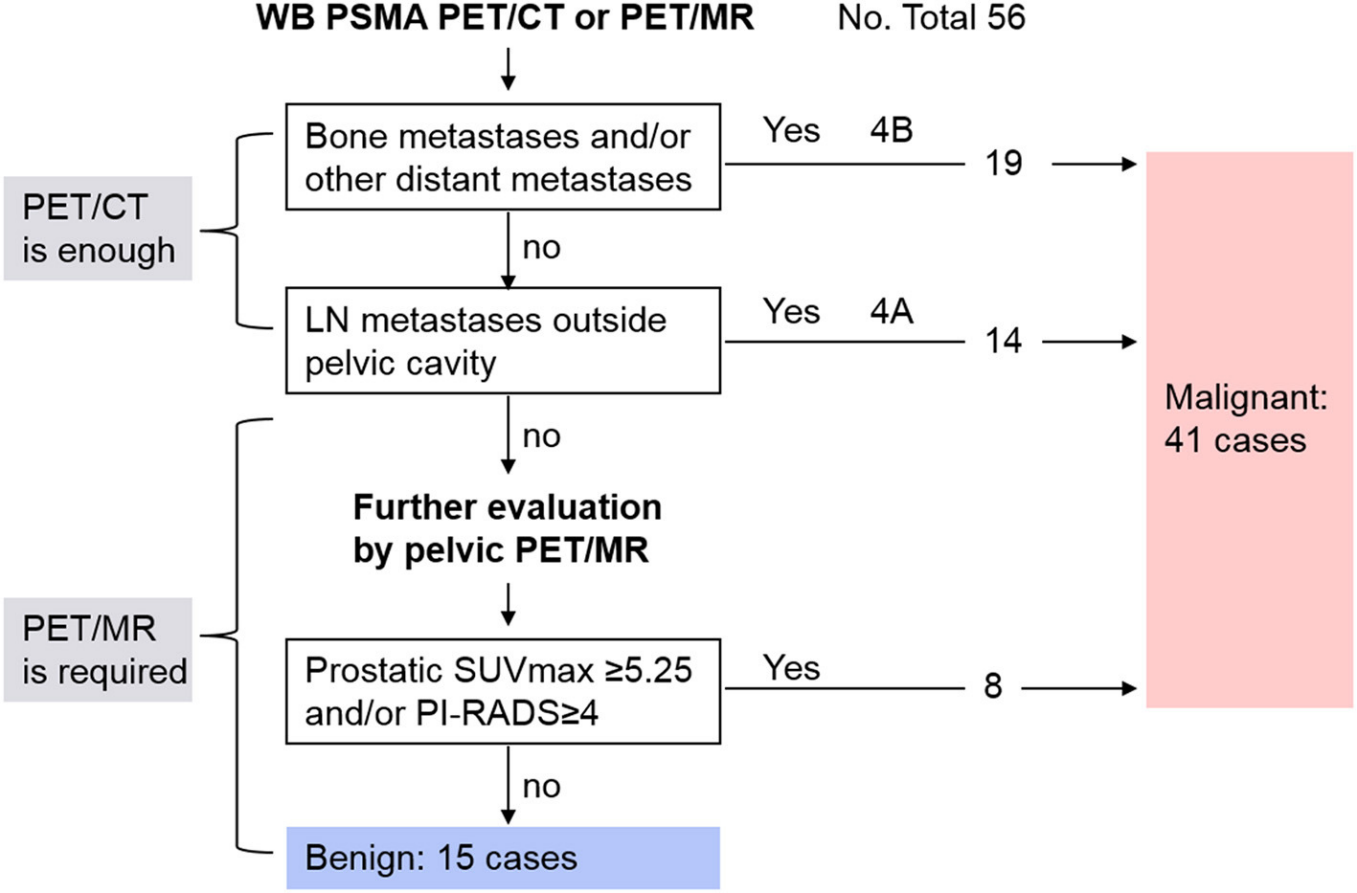
Diagnostic overview of all patients in this study. LN, lymph node.

A diagnostic overview of all patients is shown in Figure 5. Thirty-three patients at advanced stages were diagnosed and staged by conventional whole-body PET/CT or PET/MR due to detection of bone metastases and involved lymph nodes, so pelvic PET/MR imaging of these patients would be redundant. Among the rest of the patients, delayed pelvic PET/MR was used for further evaluation. Seven had prostatic foci with SUVmax > 5.25 (Figure 2A), and 1 had a PI-RADS score ≥ 4 and SUVmax <5.25 (Figure 2B), which were diagnosed as malignant and accurate staging was identified by invasion of the capsule and/or surrounding tissue provided by MR. The remaining 15 patients were diagnosed with benign disease.

## Discussion

This retrospective study confirmed the value of PSMA PET/CT and PET/MR in prostate disease diagnosing and staging. In this study, whole-body PET/CT (PET/MR) and delayed pelvic PET/MR accurately diagnosed and staged all of the patients. When the SUVmax from a conventional scan was used as the only criterion, sensitivity and specificity reached 85.4% and 100%, respectively. Our optimal cutoff value is 5.25, which is close to literature reports of 5.94 (23). However, PSMA uptake in the prostate is not significantly elevated in some patients; fortunately, nodal or bone metastases were observed on PET/CT or PET/MR imaging (Figure 1), so combination of SUVmax with nodal or bone metastases further improved the sensitivity to 95.1%. There are still a few cases with neither bone, nodal metastasis nor positive prostatic PSMA uptake. In this situation, mpMRI may improve detection (Figure 2B). Combination use of nodal metastases, bone metastases, SUVmax of primary lesions, and MR PI-RADS score from whole-body PET/CT and PET/MR made the sensitivity and specificity reach 100% for prostate diseases diagnosis in this study.

Because it has been proven that the FDG SUV measured by PET/CT and PET/MR has clinically acceptable repeatability (24, 25), our PET/CT and PET/MR scanner are calibrated to ensure the accuracy of the measurements. We ignored the influence of SUV from different devices for statistics. Our results showed that SUVmax values from delayed scans were significantly higher than those from conventional scans in patients with prostate cancer, indicating tracer accumulation increased overtime. Park, S. Y. reported similar results (26). Our results revealed SUVmax values from conventional scanning at about 1 h post injection and those from delayed scan at about 3 h are linearly correlated, which is consistent with the results reported by Ringheim et al. (27). However, they also found a mean 20% difference between PET/CT than on PET/MR (higher on PET/CT), which cannot be used interchangeably in follow-up (27). The difference between PET/CT and PET/MR devices may affect image quality and SUV measurement, therefore, a more detailed study is needed to evaluate the quantitative accuracy of PET/MR and the factors governing it.

As for the optimal timing for prostate PSMA imaging, ROC analysis shows that SUVmax1 from conventional imaging has relatively good diagnostic performance compared with delayed imaging, so we believe that conventional imaging at about 1 h is sufficient for most patients. Delayed imaging takes up time, increases patient anxiety, and may make patients with urinary retention uncomfortable, so it is not necessary on a routine basis. However, for those patients who are difficult to diagnose and whose pelvic images were affected by urine in the bladder, it is necessary to perform delayed imaging after drinking plenty of water and urinating. Some studies have analyzed multiple time-point ^68^Ga-PSMA imaging, including early dynamic images, static scans after 60 min (conventional scan) and 180 min (delayed scan) post-injection. Kabasakal et al. (28) and Uprimny et al. (29) demonstrated that early PET/CT pelvic imaging has better lesion detectability of lesions in the pelvis than in late images because of the low incidence of halo artifact in the bladder. Another study showed that PET/MR early acquisition has high lesion contrast with very good agreement for lesion detectability with same-day whole-body PET/CT. However, 95% LOAs in SUVpeak and Metabolic Tumor Volume (MTV) are far beyond the clinically acceptable range. Therefore, they suggested whole-body PET/CT and early PET/MR should not be used interchangeably (19). Some studies compared conventional and delayed scans, and reported that detection rates were the same between 60 and 180 min, although improved contrast and an additional cancer focus was found, they concluded that delayed imaging has limited impact (26, 30). However, Afshar-Oromieh et al. (31) reported different results: compared with 1 h after injection, 3-h images revealed higher detection rates and more lesions, but these PET-positive lesions were not confirmed by histopathology.

PSA has a certain value for the identification of benign and malignant prostate lesions (3). This study showed that the difference of fPSA/tPSA between benign and malignant groups is not statistically significant, while the differences of tPSA and fPSA between the two groups are statistically significant, but there are false positive and false negative results and the sensitivity and specificity are not as good as PSMA PET imaging. One study confirmed that SUVmax correlated significantly with PSA level (26). However, our results detected no correlation between tPSA, fPSA and SUVmax, while fPSA/tPSA was negatively correlated with SUVmax. Incomplete data maybe one reason and further study is needed.

Gleason score is a commonly used grading method for prostate cancer. However, we could not find any correlation between SUVmax of primary tumor and Gleason scores. The same results were also reported in two studies, due to inherent bias of the limited range of Gleason scores (23, 32). Our results also showed lymph node and distant metastases presented in patients with low Gleason scores, so Gleason score cannot reflect clinical stage, the possible reason may due to underestimate of the actual Gleason score from biopsy in some patients. These results suggest that imaging techniques have better performance in the detection of prostate cancer than screening techniques such as PSA, DRE, and TRUS-guided biopsy.

Despite many studies reporting the advantages of PET/MR (26, 33–35), its limitations include high expense, a relatively long whole-body scanning time, low visibility of lung lesions, and challenges in patients with claustrophobia and metal implants. For 33 patients with advanced prostate cancer, PET/CT is enough because these patients have nodal and/or distant metastases which can be easily diagnosed by PET/CT only, and PET/CT is better for lung lesion detection, and more time-saving and economic than PET/MR, so pelvic PET/MR had no incremental value. However, to accurately stage those patients without obvious lymph node or bone involvement, to differentiate BPH or prostatitis from early stage or PSMA-negative prostate cancer, pelvic PET/MR is required mainly because of the high soft tissue resolution of MR (20). For 7 patients with SUVmax ≥ 5.25, PET/MR identified whether there is invasion of the capsule and surrounding tissue, which defined T stages of disease precisely. For the 16 patients with SUVmax <5.25, PET/MR identified one patient with PI-RADS ≥ 4 and diagnosed as prostate cancer, playing a decisive role. In the other 15 patients with benign disease, MR-based PI-RADS crosschecked with PET results to enhance diagnostic confidence. Thus, PET/MR actually played role in 43.4% (23/53) of the patients in this study. As reported, PI-RADS 3 lesions are difficult to diagnose by MR only; PET/MR improves the detection of these patients through PET (36). Therefore, whole-body PET/CT with or without pelvic PET/MR would be a sufficient, time-saving method for initial diagnosis and accurate staging of prostate cancer, rational use of pelvic PET/MR for proper patients is important. In centers with both PET/CT and PET/MR, we recommend that all patients undergo PET/CT scanning first, and if lymph node and/or bone metastases are found, the patient can be diagnosed as advanced prostate cancer and PET/MR is unnecessary. Otherwise, according to whether the image quality and prostatic lesion detectability were affected by activity of ^68^Ga-PSMA from urine in bladder, pelvic PET/MR should be performed subsequently or at about 3 h post injection to further evaluate the prostate, surrounding tissue involvement, and small lymph nodes (Figure 2C), if the patient has no contraindications. Undoubtedly, PET/MR provides superior diagnostic performance in local prostate cancer recurrence, especially biochemical failure, compared with ^68^Ga-PSMA-617 PET/CT (37–39).

There are some limitations to the present study. First, the number of cases, especially the patients without disseminated disease, is relatively limited. Second, due to it being a retrospective study, some patients did not have a histopathology confirmation and Gleason score, they were diagnosed through comprehensive clinical evaluation and follow-up after treatment. Third, some data are not suitable for statistical analysis, such as the results of tPSA > 100 ng/L, fPSA > 30 ng/L in some patients. Fourth, the high proportion of advanced prostate cancer in this study may have affected the final results. Therefore, larger sample-sized study with early-stage patients should be included to further evaluate the incremental value of PET/MR.

## Conclusion

This study confirmed that a combination application of PSMA whole-body PET/CT and pelvic PET/MR can accurately distinguish BPH/prostatitis from prostate cancer and accurately stage prostate cancer. Whole-body PET/CT is sufficient to diagnose advanced prostate cancer. The value of pelvic PET/MR is for the diagnosis and accurate staging of early prostate cancer. Conventional imaging at about 1 h is recommended with no need to perform delayed imaging for most patients. Further study with more early-stage prostate cancer patients and a prospective design is needed.

## Data Availability Statement

The raw data supporting the conclusions of this article will be made available by the authors, without undue reservation.

## Ethics Statement

The studies involving human participants were reviewed and approved by Human Ethics Committee of Union hospital, Tongji Medical College, Huazhong University of Science and Technology. The patients/participants provided their written informed consent to participate in this study.

## Author Contributions

CQ conducted the analyses and was a major contributor in writing the manuscript. YG and QL synthesized the radiopharmaceuticals and performed quality control. WR and FH acquired PET/CT and PET/MR images. FL analyzed the images. XZ provided clinical information of all patients and contributed to revision. XL conceived the idea and contributed to analysis and revision. All authors read and approved the final manuscript.

## Conflict of Interest

The authors declare that the research was conducted in the absence of any commercial or financial relationships that could be construed as a potential conflict of interest.
